# Supplementary material for: Fish Community Structure and Biomass Particle-Size Spectrum in the Upper Reaches of the Jinsha River (China)
Source: Animals (Basel). 2022 Dec 4;12(23):3412. doi: 10.3390/ani12233412 (PMC9739501; doi:10.3390/ani12233412)
Supplement: Supplementary file 1 [file animals-12-03412-s001.zip › animals-1981835-supplementary.pdf]

animals

MDPI

Table S1. Fish species in the Upper reaches of the Jinsha River.

Species	Ecological type	Wet Season	Dry season	Hu [6]	Zhang *et al* [7]
1. *Oreias dabryi**	O; SE		+	+	+
2. *Triplophysa brevicauda*	O; SE		+	+	+
3. *Triplophysa leptosome*	O; SE				+
4. *Triplophysa stoliczkae*	O; SE	+	+	+	
5. *Triplophysa stenura*	O; SE	+	+	+	
6. *Triplophysa angeli*	O; SE		+	+	
7. *Trilophysa bleekeri*	O; SE	+	+	+	
8. *Triplophysa yaopeizhii**	O; SE		+	+	
9. *Misgurnus anguillicaudatus*△	O; SE	+			
10. *Paramisgurnus dabryanus*△	O; SE		+		
11. *Pseudorasbora parva*△	O; SE	+			
12. *Ctenopharyngodon idellus*△	H; SH				+
13. *Abbottina rivularis*△	O; SE	+			
14. *Cyprinus carpio*△	O; SE	+	+		
15. *Carassius auratus*△	O; SE	+	+		
16. *Schizothorax wangchiachii**	O; SH	+	+	+	+
17. *Schizothorax prenanti**	O; SH	+	+		
18. *Schizothorax grahami**	O; SH				+
19. *Schizothorax chongi**	O; SH	+			
20. *Schizothorax dolichonema**	O; SH	+	+	+	+
21. *Schizothorax kozlovi**	O; SH	+	+	+	+
22. *Ptychobarbus kaznakovi*	O; SH	+	+	+	
23.*Gymnodiptychus pachycheilus*	O; SH	+	+		+
24. *Gymnocypris potanini**	O; SH	+	+		+
25. *Schizopygopsis malacanthus**	O; SH	+	+	+	+
26. *Herzensteinia microcephalus**	O; SH		+	+	
27. *Jinshaia sinensis**	O; SE	+	+		+
28. *Lepturichthys fimbriata**	O; SE				+
29. *Silurus meridionalis*△	O; SE	+			
30. *Clariidae gariepinus*△	C; SE				+
31. *Euchiloglanis kishinouyei**	C; SE	+	+	+	+
32. *Euchiloglanis davidi**	C; SE	+			
33. *Pareuchiloglanis anteanalis*	O; SE	+	+		+
34. *Pareuchiloglanis sinensis**	O; SE			+	+
35. *Micaopercops swinhonis*△	C; SE				+

Note:*-endemic species of the Jinsha River; △-alien species; C-carnivorous; H-phytophagy; O-omnivory; SH-short migration; SE-settled fishes.
